# Pre-Pregnancy Excess Weight Association with Maternal Sociodemographic, Anthropometric and Lifestyle Factors and Maternal Perinatal Outcomes

**DOI:** 10.3390/nu14183810

**Published:** 2022-09-15

**Authors:** Dimitrios Papandreou, Maria Mantzorou, Stefanos Tyrovolas, Eleni Pavlidou, Georgios Antasouras, Evmorfia Psara, Efthymios Poulios, Georgios K. Vasios, Constantinos Giaginis

**Affiliations:** 1 Department of Health Sciences, College of Natural and Health Sciences, Zayed University, Abu Dhabi P.O. Box 144534, United Arab Emirates; 2 Department of Food Science and Nutrition, School of Environment, University of the Aegean, 81400 Myrina, Lemnos, Greece; 3 Department of Nursing, The Hong Kong Polytechnic University, Kowloon, Hong Kong; 4 Research, Innovation and Teaching Unit, Parc Sanitari Sant Joan de Déu, 08830 Sant Boi de Llobregat, Spain; 5 Instituto de Salud Carlos III, Centro de Investigación Biomédica en Red de Salud Mental, CIBERSAM, 28029 Madrid, Spain

**Keywords:** obesity, overweight, pregnancy, maternal risk factors, perinatal outcomes

## Abstract

Background: Pre-pregnancy excess weight is an important factor for adverse maternal perinatal outcomes; however, data for Greek women remain limited. Therefore, the aim of the present work was to evaluate the relation between pre-pregnant weight status and sociodemographic, anthropometric and lifestyle factors and maternal perinatal outcomes. Methods: In the present cross-sectional study, 5133 healthy women were enrolled from nine different Greek regions after applying specific inclusion and exclusion criteria. Validated questionnaires were used to assess the sociodemographic characteristics and certain lifestyle factors of the study population. Anthropometric and clinical data were retrieved from medical history files of the women, including measured weight in the first weeks of pregnancy and right before delivery, and maternal perinatal outcomes. Women’s weights and heights were also measured 2–5 years postpartum by trained nutritionists. Non-adjusted and adjusted statistical analysis was performed to assess whether pre-pregnancy weight status was associated with sociodemographic, anthropometric and lifestyle factors and maternal perinatal outcomes. Results: In pre-pregnancy, 17.5% of the women were overweight, and 4.9% were classified as obese. These rates were increased 2–5 years postpartum, reaching 21.0% for overweight and 9.6% for obese women. Pre-pregnancy overweight/obesity were associated with older maternal age, higher prevalence of overweight/obesity at 2–5 years postpartum and nonexclusive breastfeeding, as well as increased rates for preterm birth and pregnancy-induced hypertension after multiple adjustments. Conclusions: Overweight and obesity rates were high among women of childbearing age in Greece. These findings highlight the urgent need for healthy lifestyle promotion and targeted obesity prevention and intervention schemes among women of reproductive age.

## 1. Introduction

Gestational weight gain, gestational diabetes, pregnancy-induced hypertension and preterm birth are highly prevalent among women in developed countries [1]. At a physiological level, the aforementioned adverse health outcomes have mutual connections that are related to placental vascular development [2]. In addition, various socioeconomic (i.e., education level) and lifestyle risk factors (i.e., smoking, physical inactivity and dietary patterns) [3] have been found to be associated with increased rates of maternal adverse health outcomes especially during the perinatal period [4,5,6]. Furthermore, underreporting or under-registration of maternal health during the perinatal and postpartum periods still exists in industrialized countries [7].

Understanding the impact of modifiable risk factors among women of childbearing age is crucial for developing strong pre- and post-pregnancy prevention policies and effective monitoring systems. One of the modifiable risk factors is excess weight before, during, and after pregnancy [8]. Specifically, pre-pregnancy obesity has been identified as a modifiable risk factor for the development of maternal perinatal outcomes [9]. In fact, maternal obesity has been correlated, in different populations, with an increased risk of health outcomes such as gestational hypertension, diabetes, preterm birth, macrosomia, and caesarean delivery [10]. In addition, few studies have shown that pre-pregnancy excess weight is correlated with weight gain at postpartum that is linked with metabolic and cardiovascular diseases later on [11]. The prevalence of overweight and obesity among women of reproductive age has been recorded at alarming rates for developed countries. Recent obesity data show that these figures are around 20% and more for the US [12], while an average prevalence of 12–14% has been recorded for European Union (EU) countries. World Health Organization (WHO) 2022 estimates that more than 20% of women in some European countries, such as Spain and Hungary, are obese when they become pregnant [13]. In Greece, data from a pilot study reported figures close to the average aforementioned rates (20% overweight and 7% obese before pregnancy) [14]. To date, the relevant information for Greece is still limited. Assessment of pre-pregnancy excess weight and its correlation with maternal perinatal health outcomes and with the postpartum period is an important step in order to shape any future prevention programs among European/Mediterranean countries as well as Greece, where current information is lacking. In addition, recent systematic reviews and meta-analyses have shown that physical activity and weight loss interventions as well as dietary interventions during pre-pregnancy or pregnancy may be beneficial for preventing and/or decreasing adverse perinatal health outcomes [15,16].

Given the increasing prevalence of obesity in the Mediterranean region and specifically Greece, especially among younger women of childbearing age in conjunction with the increased rates of perinatal and postpartum maternal adverse health outcomes and the limited information on them in the region, it is important to determine the role of excess weight during pre-pregnancy as well as the role of sociodemographic, anthropometric and lifestyle factors in maternal perinatal and health outcomes among Greek women. Therefore, the aim of the present work was to evaluate the correlation between pre-pregnant weight status and sociodemographic, anthropometric and lifestyle factors and maternal perinatal outcomes in a nationally representative population in Greece.

## 2. Materials and Methods

### 2.1. Subjects

In the present study, 7191 women were initially enrolled from nine different Greek regions, namely Athens, Thessaloniki, Larisa, Patra, Alexandroupolis, Kalamata, Ioannina, Crete and North Aegean. The inclusion criteria for the initial enrollment were: (a) women who had a singleton birth 2–5 years before enrollment, independently of parity, and (b) women who had no pregnancy in the interval between the time of this singleton birth and the time of the study, i.e., 2–5 years postpartum, while in multiparous women, only the last pregnancy was considered. All subjects were recruited to the study between May 2016 and September 2020. All participants’ information was confidential, and all participants were disease-free and informed about the purpose of the study and signed a consent form. Sample size calculation was based on the use of the PS: Power and Sample Size calculator program, while randomization was carried out with the use of a sequence of random binary numbers (i.e., 001,110,110, in which 0 represented enrolled, and 1 not enrolled in the study). Among 7191 initially enrolled women, 753 women (10.5%) were excluded from the study due to missing or incomplete data, resulting in a final response rate of 89.5%. Among the remaining 6438 women, 1305 (20.3%) were then excluded from the study due to any history of disease such as diabetes, hypertension, anemia, hyperlipidemia, osteoporosis, multiple sclerosis, cancer, polycystic ovary symptoms, irritable bowel syndrome, constipation, inflammatory bowel disease (ulcerative colitis and Crohn’s disease), gallstones, autoimmune liver disease, celiac disease, and pelvic floor dysfunction. A total of 5133 women were included in the final analysis. The study was approved by the Ethics Committee of the University of the Aegean (ethics approval code: no 12/14.5.2016), and was in compliance with the World Health Organization (52nd WMA General Assembly, Edinburgh, Scotland, 2000). The exclusion criterion was any disease for enrolled women except for gestational diabetes and pregnancy-induced hypertension.

### 2.2. Study Design

At the time of study, i.e., 2–5 years after delivery, validated questionnaires were used to assess the sociodemographic characteristics and lifestyle factors of the study population [3]. Women’s weight in the first weeks of pregnancy and right before delivery were retrieved from their personal gynecologists’ or hospitals’ medical files in which measured weight data had been recorded during visits to public or private health care units. Gestational weight gain was calculated by subtracting the retrieved measured weight in the first weeks of pregnancy from the retrieved measured weight right before delivery. At the time of study, the women’s weights and heights were also measured by a trained nutritionist as per protocol [3]. Weight was measured using the same electronic scale, and height was measured using a portable stadiometer.

Smoking habit, educational and economic level, and parity status were from questionnaires 2–5 years postpartum based on the women’s memory recall. In fact, educational level was scaled according to the sum of years of education, and economic status was classified according to the annual family income (in EUR) as: 0 ≤ 5000, 1 ≤ 10,000, 2 ≤ 15,000, 3 ≤ 20,000, 4 ≤ 25,000 and 5 ≥ 30,000. Financial status was further categorized as low for annual family income ≤ EUR 10,000, medium for annual family income ˃ EUR 10,000 and ≤ EUR 20,000, and high for annual family income ˃ EUR 20,000. Additionally, mothers were asked whether they breastfed their children, whether they were exclusively breastfeeding for at least 4 months and the duration of breastfeeding. To overcome recall bias, the women were asked about exclusive breastfeeding for at least 4 months because at this time point, most of them were advised to gradually introduce pulp foods to the feeding routine for their children, and they, therefore, remembered more precisely this time point, rendering their answers more reliable. In contrast, mothers who breastfed for shorter periods were not able to answer with absolute confidence about the exact duration of breastfeeding. Mothers were also asked to report if they had a preterm birth (<37th week), and their answers were further cross-checked by their gynecologists’ or hospitals’ medical files for more precise records for the exact week of preterm birth to be obtained; however, we observed that there were several missing data concerning the exact week of preterm birth, and thus, preterm birth (<37th week) was treated as a binary outcome. Women’s histories of gestational weight gain and pregnancy-induced hypertension were also retrieved from their personal gynecologists’ or hospitals’ medical files.

Clarifying instructions were given to the participants by registered dietitians regarding completion of the questionnaires, while a detailed presentation of the questions to facilitate accurate answers was performed.

#### Statistical Analysis

Statistical analysis was performed by Student’s t-test and one-way ANOVA for continuous variables found to follow the normal distribution by the use of Kolmogorov–Smirnov test. The Chi-square test was used for categorical variables, and the Mann–Whitney non-parametric test was used for non-normally distributed continuous variables between two groups, while the Kruskal–Wallis non-parametric test was applied for non-normally distributed variables between three or more groups. The normally distributed quantitative variables are presented as mean value ± standard deviation (SD), and the qualitative variables as absolute or relative frequencies. The non-normally distributed quantitative variables are presented as median value (interquartile range; IQR). Multivariate logistic regression analysis was performed to assess whether pre-pregnancy body mass index (BMI) status was independently associated with maternal sociodemographic, anthropometric and lifestyle factors and maternal perinatal outcomes after adjustment for potential confounders, e.g., women’s age, nationality, educational and economic status, smoking habits, gestational weight gain, exclusive breastfeeding, parity, preterm birth, gestational diabetes and pregnancy-induced hypertension. Differences were considered significant at *p* < 0.05 and 95% confidence interval. The statistical analysis of the survey data was performed in the SPSS 21.0 program (Statistical Package for Social Sciences, Chicago, IL, USA).

## 3. Results

### 3.1. Sociodemographic, Anthropometric and Lifestyle Characteristics and Maternal Perinatal Outcomes of the Study Population

The present study finally included 5133 healthy women who were enrolled 2–5 years after delivery. Mean age of the women was 37.5 ± 4.8 years. Concerning nationality, 95.8% of the women were Greek and the rest (4.2%) were of other nationalities (mainly Russian, Albanian, Ukrainian, Bulgarian and Romanian). As far as the educational level of the mothers was concerned, the mean years of education was 15.1 ± 2.2 years (range: 6–17 years of education). With regard to the economic level, 46.1% of the participant women reported low annual family income, while 45.2% and 8.7% reported medium and high annual family income, respectively. Moreover, 25.6% of the women were smokers both during pre-pregnancy and 2–5 years after delivery.

The mean BMI of the mothers before pregnancy was 22.7 ± 3.7 kg/m^2^ (range: 15.9–37.6 kg/m^2^), while at the time of the study (2–5 years postpartum), the mean BMI was significantly higher, at 23.7 ± 4.4 kg/m^2^ (range: 16.3–41.5 kg/m^2^, *p* < 0.0001). More to the point, in pre-pregnancy, 17.5% of the women were overweight, and 4.9% were obese, according to their BMI. In total, a prevalence of 22.4% was recorded concerning pre-pregnancy overweight and obesity of women in the study. At the time of study (2–5 years postpartum), 21.0% of the women were overweight, and 9.6% were obese, according to their BMI. Thus, the prevalence of both overweight and obesity were considerably increased 2–5 years postpartum, reaching 30.6%. The incidence of underweight women was very small both in pre-pregnancy (3.6%) and 2–5 years postpartum (3.9%) and did not differ significantly; thus, underweight women were included in the normal weight group without affecting the following analysis. In addition, an initial analysis where underweight women were treated as a single category was performed, which showed no confounding effect on the associations of pre-pregnancy overweight/obesity with the examined characteristics.

Half of the women (50.4%) did breastfeed exclusively for at least 4 months (mean duration: 4.8 ± 1.9 months), and 49.6% did not exclusively breastfeed for at least 4 months, or did not breastfeed at all. Of the study population, 26.7% did not breastfeed at all. Concerning parity, 64.3% of the women reported that they were nulliparous (no previous birth), and the remaining 35.6% were multiparous (at least one previous birth).

The mean gestational weight gain was 13.8 ± 6.1 kg (range 4.0–45.0 kg). A prevalence of preterm birth (<37th week) equal to 30.2% of the study population was recorded. Moreover, 4.3% of the women developed gestational diabetes mellitus during their pregnancy, and 4.2% of the women developed pregnancy-induced hypertension.

### 3.2. Pre-Pregnancy Overweight and Obesity in Association with Sociodemographic, Anthropometric and Lifestyle Characteristics of the Participant Women

**Figure 1:**
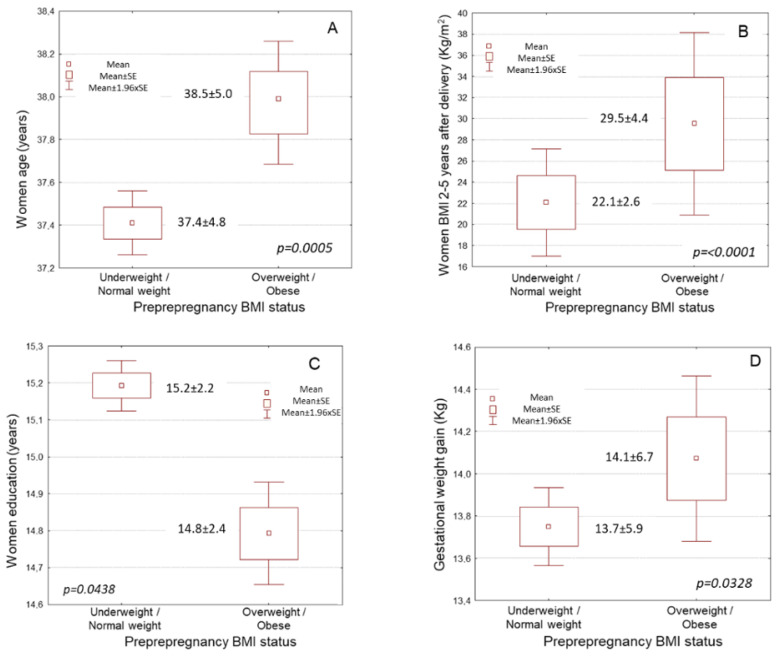
Associations of pre-pregnancy overweight and obesity with women: (**A**) Age, (**B**) Educational level, (**C**) BMI 2–5 years after delivery and (**D**) Gestational weight gain. BMI, body mass index; SE, standard error.

Pre-pregnancy overweight and obese women were significantly older compared to underweight and normal weight women (Figure 1A, Table 1, 38.5 ± 5.0 vs. 37.4 ± 4.8 years, *p* = 0.0005). In crosstabulation, Greek women showed a significantly lower incidence of pre-pregnancy overweight and obesity than women of other nationalities (Table 1, *p* < 0.0001). Pre-pregnancy overweight and obese women had significantly higher BMI values at 2–5 years after delivery compared to underweight and normal weight women (Figure 1B, Table 1, 29.5 ± 4.4 vs. 22.1 ± 2.6 kg/m^2^, *p* < 0.0001).

Furthermore, those who were overweight or obese pre-pregnancy had marginally lower educational levels than those who had underweight or normal BMI pre-pregnancy (Figure 1C, Table 1, 14.8 ± 2.4 vs. 15.2 ± 2.2 years *p* = 0.0438). The economic level of pre-pregnancy overweight and obese women was also significantly lower than that of women who had underweight or normal BMI pre-pregnancy (Table 1, *p* = 0.0001). Similarly, pre-pregnancy overweight and obese women were smokers at higher rates than women who had underweight or normal BMI pre-pregnancy (Table 1, *p* = 0.0029). Moreover, pre-pregnancy overweight and obese women had a significantly higher prevalence of multiparity compared to underweight and normal weight women pre-pregnancy (Table 1, *p* = 0.0082).

### 3.3. Pre-Pregnancy Overweight and Obesity in Association with Maternal Perinatal Outcomes

Pre-pregnancy overweight and obese women developed slightly higher gestational weight gain compared to pre-pregnancy underweight and normal weight women (Figure 1D, Table 1, 14.1 ± 6.7 vs. 13.7 ± 5.9 kg, *p* = 0.0328). Preterm birth was significantly more frequently observed in pre-pregnancy overweight and obese women compared to underweight and normal weight women (Table 1, *p* < 0.0001). Gestational diabetes was significantly more frequently observed in pre-pregnancy overweight and obese women compared to underweight and normal weight women (Table 1, *p* = 0.0409). Moreover, pre-pregnancy overweight and obese women had a significantly higher prevalence of pregnancy-induced hypertension compared to underweight and normal weight women (Table 1, *p* = 0.0048).

### 3.4. Multivariate Regression Analysis for Pre-Pregnancy BMI Status

In the multivariate logistic regression analysis, pre-pregnancy overweight and obesity was independently associated with women’s age, BMI at 2–5 years after delivery, exclusive breastfeeding, preterm birth and pregnancy-induced hypertension after adjustment for potential confounders (Table 2, *p* < 0.05). In fact, older women (≥37.5 years old) had a 54% higher prevalence of overweight or obese before pregnancy than younger women (<37.5 years old) (Table 2, *p* = 0.0022). Pre-pregnancy overweight and obese women showed a more than two-fold higher risk of being overweight or obese at 2–5 years after delivery than underweight and normal weight women (Table 2, *p* = 0.0001). Pre-pregnancy overweight and obese women also had 35% lower odds of following exclusive breastfeeding after delivery than underweight and normal weight women (Table 2, *p* = 0.0082).

Pre-pregnancy overweight and obese women had a 44% higher probability of preterm birth compared to underweight and normal weight women pre-pregnancy (Table 2, *p* = 0.0044). Moreover, a 34% higher likelihood for developing pregnancy-induced hypertension was observed in pre-pregnancy overweight and obese women compared to underweight and normal weight women (Table 2, *p* = 0.0231). Women’s nationality, education and economic level, smoking habits, gestational weight gain, parity and gestational diabetes were not associated with pre-pregnancy BMI status in the multivariate analysis (Table 2, *p* > 0.05).

## 4. Discussion

The present work revealed that pre-pregnancy overweight and obesity are highly prevalent among Greek women, while economic and education status were among the indicators of pre-pregnancy overweight and obesity epidemiology. Pre-pregnancy overweight and obesity were independently correlated with a range of maternal perinatal outcomes (i.e., increased postpartum BMI). The results are indicative of the need for public health actions such as prevention of obesity among women of childbearing age in Greece, in order to improve maternal perinatal health and postpartum weight retention that could possibly lead to metabolic and cardiovascular disease in mothers later in life.

The prevalence of pre-pregnancy overweight and obesity was high among the women in our sample, while education and economic status were among the related predictors. This observation is in line with some previous pilot data for Greece and Europe [4,14]. Furthermore, pre-pregnancy overweight and obesity were correlated with higher BMI retention at 2–5 years postpartum, a finding that was also in line with previous studies [17], and that has been shown to affect maternal metabolic and cardiovascular health [18]. Pre-pregnancy obesity prevention and postpartum weight retention have been noted recently by international organizations [19], as emerging new public health challenges due to their correlation with both short- and long-term adverse maternal and offspring outcomes [18]. These results—in combination with the obesity epidemic in Greece [20,21]—raise major concerns about the need for early obesity prevention measures in pre-pregnancy stages as well as for intervention measures during the postpartum period, targeting specifically groups of low socioeconomic status.

Furthermore, the association of pre-pregnancy overweight and obesity with perinatal maternal outcomes was evaluated, taking into account several confounders. It was shown that pre-pregnancy overweight and obesity were correlated with increased odds of the mother retaining her weight at postpartum, as well as increased odds of gestational weight gain, preterm birth, gestational diabetes, and pregnancy-induced hypertension. In addition, it was found that pre-pregnancy overweight and obesity were inversely associated with exclusive breastfeeding. Previous data have indicated that pre-pregnancy weight could be independently correlated with perinatal maternal health outcomes [3,22]. However, to the best of our knowledge, this is one of the first large-scale studies to report such results for the regions of Greece. Identification of these factors is very important, as there is a lack of research in the area and underreporting [7], and such data [23] could help to shape future prevention and monitoring program for maternal health, battling obesity before, during, and after pregnancy in Greece and the southern Mediterranean region.

Recent studies have shown that physical activity as well as healthy dietary patterns (such as the Mediterranean diet) are correlated with beneficial perinatal outcomes [5,24]. Stakeholders in Greece could consider excess weight among the targeted risk factors for the country’s women of childbearing age. Maintaining normal weight through physical activity and specific dietary habits within the Mediterranean diet or other healthy dietary patterns could serve as cost-effective actions for maternal disease prevention [22]. Of course, future studies should evaluate in depth physical activity and nutritional and other lifestyle interventions to attain a beneficial weight status among Greek women and potentially decrease the risk on adverse maternal and child health outcomes.

The present study has several strengths. It is one of the few, especially for Greece, to evaluate pre-pregnancy excess weight and the related risk factors as well as their effects on maternal perinatal health outcomes in a nationally representative sample of nine geographically diverse areas of our country. In terms of limitations, the cross-sectional nature of the study limits the possibility of etiological conclusions and also has the potential for recall biases, especially for self-reported responses to questions. BMI was used to define maternal overweight and obesity; however, direct measures of body fat mass and distribution are needed to extend and confirm our findings. Moreover, even though we adjusted for multiple confounding factors, it is still possible that residual confounding may have affected our results. Additional lifestyle or health-related factors, such as physical activity, dietary habits and mental health status, may confound the influence of pre-pregnancy overweight/obesity in the adjusted statistical model, and thus, future studies should take into account these factors.

## 5. Conclusions

Overweight and obesity rates were high among women of childbearing age in Greece. Moreover pre-pregnancy excess weight was correlated with increased postpartum BMI among women and also was associated with several adverse perinatal maternal outcomes. These results point to the importance of primary and secondary prevention efforts, especially for obesity during pre-pregnancy and postpartum periods, in reducing a variety of adverse perinatal maternal outcomes among Greek women. Taking into account the fact that obesity among women of childbearing age poses a public health challenge, especially in Greece and other Mediterranean countries, the aforementioned findings highlight the urgent need for healthy lifestyle promotion and targeted obesity interventions among women of reproductive age.

## Figures and Tables

**Table 1:** Associations of pre-pregnancy BMI status with mothers’ sociodemographic and anthropometric characteristics, and maternal outcomes.

Parameters (*n* = 5133)	Pre-Pregnancy BMI Status		
	Underweight and Normal Weight (77.6%)	Overweight and Obese (22.4%)	*p*-Value
Age (years)	37.4 ± 4.8	38.5 ± 5.0	*p* = 0.0005
Nationality (%)			*p* < 0.0001
Greek	3843 (95.5)	1070 (92.9)	
Others	138 (3.5)	82 (7.1)	
BMI 2–5 years after delivery (kg/m^2^)	22.1 ± 2.6	29.5 ± 4.4	*p* < 0.0001
BMI 2–5 years after delivery (%)			*p* < 0.0001
Underweight	193 (4.9)	11 (0.9)	
Normal weight	3270 (82.1)	89 (7.7)	
Overweight	475 (11.9)	602 (52.3)	
Obese	43 (1.1)	450 (39.1)	
Education (years)	15.2 ± 2.2	14.8 ± 2.4	*p* = 0.0438
Economic status (%)			*p* = 0.0001
Low	1783 (44.8)	581 (50.4)	
Medium	1814 (45.6)	508 (44.1)	
High	384 (9.6)	63 (5.5)	
Smoking habits (%)			*p* = 0.0029
Nonsmokers	3000 (75.4)	818 (71.0)	
Smokers	981 (24.6)	334 (29.0)	
Gestational weight gain (kg)	13.7 ± 5.9	14.1 ± 6.7	*p* = 0.0328
Exclusive breastfeeding (%)			*p* < 0.0001
No	1849 (46.4)	735 (63.8)	
Yes	2132 (53.6)	417 (36.2)	
Parity (%)			*p* = 0.0082
Nulliparity	2598 (65.3)	703 (61.0)	
Multiparity	1383 (34.7)	449 (39.0)	
Preterm birth (<37th week, %)			*p* < 0.0001
No	2858 (71.8)	726 (63.0)	
Yes	1123 (28.2)	426 (37.0)	
Gestational diabetes (%)			*p* = 0.0409
No	3822 (96.0)	1090 (94.6)	
Yes	159 (4.0)	62 (5.4)	
Pregnancy-induced hypertension (%)			*p* = 0.0048
No	3799 (95.4)	1121 (97.3)	
Yes	182 (4.6)	31 (2.7)	

BMI, body mass index.

**Table 2:** Multivariate logistic regression analysis of influence of women’s pre-pregnancy BMI status.

Parameters	Pre-Pregnancy Overweight and Obesity	
	HR * (95% CI **)	*p*-Value
Age (below/over mean value)	1.54 (0.95–2.20)	*p* = 0.0022
Nationality (Greek/other nationality)	1.16 (0.81–1.69)	*p* = 0.0955
BMI 2–5 year after delivery (underweight or normal/overweight or obese)	2.17 (1.89–2.38)	*p* = 0.0001
Education (below/over mean value)	0.73 (0.48–1.09)	*p* = 0.0879
Economic status (low/medium or high)	0.72 (0.31–1.26)	*p* = 0.3951
Smoking habits (No/Yes)	2.58 (1.81–3.22)	*p* = 0.2108
Gestational weight gain (below/over mean value)	1.29 (0.74–1.82)	*p* = 0.0729
Exclusive breastfeeding (No/Yes)	0.65 (0.28–1.05)	*p* = 0.0082
Parity (Nulliparity/Multiparity)	0.61 (0.28–0,97)	*p* = 0.1523
Preterm birth (No/Yes)	1.44 (0.82–2.03)	*p* = 0.0044
Gestational diabetes (No/Yes)	1.37 (0.91–1.98)	*p* = 0.0982
Pregnancy-induced hypertension (No/Yes)	1.34 (0.71–1.95)	*p* = 0.0231

* Hazard Ratio: HR; ** CI: Confidence Interval.

## Data Availability

Data available upon request.
